# Bridging trust and performance in intelligent systems: Hybrid explainable AI approaches for interpreting large language models

**DOI:** 10.1371/journal.pone.0343472

**Published:** 2026-09-15

**Authors:** Arul Selvam P., Tamije Selvy P.

**Affiliations:** 1 Department of Artificial Intelligence and Machine Learning, Hindusthan College of Engineering and Technology, Coimbatore, Tamil Nadu, India; 2 Department of Computer Science and Engineering, Hindusthan College of Engineering and Technology, Coimbatore, Tamil Nadu, India; Federal University of Paraiba, BRAZIL

## Abstract

Large Language Models (LLMs) achieve state-of-the-art performance across natural language processing tasks but remain opaque, limiting adoption in high-stakes domains that demand accountability and transparency. This paper introduces a hybrid explainability framework that integrates saliency-based attribution, causal reasoning, and user-centered visualization into a unified, efficiency-aware pipeline. Unlike prior single-method approaches such as LIME, SHAP, or attention visualization, the framework provides explanations that are both technically faithful and accessible to human evaluators. The framework was systematically evaluated on benchmark datasets (GLUE, SQuAD, IMDB, and domain-specific corpora) and tested on representative architectures (BERT, T5, GPT, and LLaMA). Results show up to a 15–20% improvement in fidelity compared to attention-based methods. Fidelity was measured using standardized insertion and deletion metrics across all benchmark datasets using a consistent evaluation protocol, ensuring objective and comparable assessment of explanation faithfulness across different LLM architectures. The proposed framework also achieved higher clarity and trust ratings in user studies while introducing less than 25% additional computational overhead. Case studies in sentiment analysis and question answering further demonstrate that hybrid explanations produce precise, intuitive reasoning paths that outperform existing baselines. The main contributions are: (1) a multi-method pipeline that reconciles the trade-off between faithfulness and interpretability; (2) a human-centered evaluation showing hybrid explanations are more trustworthy than single techniques; and (3) an efficiency-aware design indicating the potential suitability of the proposed framework for practical applications in domains such as healthcare, finance, and law. By aligning methodological rigor with societal and regulatory demands, this study advances both the practice and theory of explainable AI, positioning hybrid XAI as a pathway toward responsible LLM adoption.

## 1 Introduction

Large Language Models (LLMs) such as GPT, BERT, and T5 have transformed natural language processing (NLP), achieving near-human performance in tasks including translation, summarization, question answering, and dialogue generation. Their cross-domain generalization has enabled applications in healthcare diagnostics, legal reasoning, financial forecasting, and decision support. However, the opacity of their internal mechanisms poses serious challenges for accountability, user trust, and operational safety, particularly in high-stakes or regulated domains [1].

Explainable Artificial Intelligence (XAI) aims to mitigate these challenges by clarifying how models arrive at predictions. Traditional XAI methods—such as feature attribution, saliency mapping, and rule extraction—have been widely applied to structured data and computer vision. Yet, extending these techniques to LLMs is non-trivial: transformer-based architectures, billions of parameters, and context-dependent token interactions complicate interpretability [1,2]. Moreover, emergent capabilities such as reasoning and knowledge generalization resist simple linear explanations, limiting the reliability of standard attribution methods [3].

The urgency for explainability has intensified with the rapid deployment of LLMs in sensitive sectors. Lack of interpretability hampers bias detection, error analysis, and human oversight. In healthcare and finance, explainability is not just desirable but a regulatory and ethical necessity [4]. Consequently, advancing explainability for LLMs is both a scientific and societal imperative.

Recent surveys categorize LLM explainability into local approaches (explaining individual predictions through token attribution or influence tracing) and global approaches (characterizing embedding spaces, probing internal representations, or analyzing training dynamics) [1,2]. Despite progress, existing methods remain limited: explanations often lack fidelity (misrepresenting actual reasoning), impose high computational costs, or fail to provide insights in a format accessible to non-technical users.

This study introduces a hybrid explainability framework for LLMs that advances beyond prior approaches by:

1. Integrating complementary methods—saliency-based attribution, causal intervention, and user-centered visualization—into a unified, efficiency-aware pipeline tailored to LLM architectures.
2. Balancing multiple dimensions of evaluation—fidelity, interpretability, and computational efficiency—rather than focusing narrowly on explanation accuracy alone.
3. Embedding human-centered usability by incorporating visualization dashboards and validating effectiveness through case studies and user evaluations, thereby extending XAI into practical decision-support contexts.

The framework is tested on benchmark NLP datasets and representative LLMs, with results demonstrating improvements in both faithfulness and usability.

**Contributions.** The major contributions of this study are:

1. A novel hybrid XAI framework that uniquely combines saliency attribution, causal reasoning, and visualization into a single, efficiency-aware design for LLM interpretability.
2. A comprehensive empirical evaluation across diverse LLMs and benchmark datasets, jointly assessing fidelity, runtime efficiency, and human comprehension.
3. Qualitative case studies and user-centered validation showing that hybrid explanations enhance trust, debugging, and bias detection in critical domains.
4. A practical roadmap for developing trustworthy and transparent LLMs aligned with emerging ethical, societal, and regulatory expectations.

The remainder of this paper is structured as follows: Section 2 reviews related work, Section 3 introduces the proposed framework, Section 4 outlines the experimental setup, Section 5 reports results, Section 6 discusses implications, and Section 7 concludes with future research directions.

## 2 Related work

This section reviews prior studies on explainability in both machine learning and large language models (LLMs) and identifies the key gaps that motivate the proposed framework. A structured comparison between conventional XAI methods and the proposed hybrid approach is presented in Table 1. Traditional methods such as LIME [5], SHAP [6], and Grad-CAM [7] provide localized or feature-level explanations but often lack scalability and causal interpretability when applied to modern LLMs. Transformer-based attention visualization offers a native form of interpretability; however, its reliability has been questioned because attention weights do not necessarily correspond to actual decision-making processes [8]. More recent studies have explored causal reasoning techniques [9,11] and interactive visualization tools [10] to improve explanation fidelity and usability. Nevertheless, these approaches remain largely isolated contributions and are rarely integrated into a unified explainability framework. In contrast, the proposed framework combines saliency attribution, causal intervention, and visualization within a single efficiency-aware pipeline, thereby bridging the gap between explanation fidelity and practical usability.

**Table 1:** Comparative summary of existing XAI methods and the proposed hybrid framework.

Approach / Method	Core Technique	Strengths	Limitations in LLMs	Relevance to Proposed Framework
LIME ([5])	Local surrogate models	Simple, model-agnostic, intuitive	Unstable results, poor scalability for LLMs	Provides baseline local interpretability
SHAP ([6])	Shapley value approximations	Theoretically grounded, consistent	High computational cost, limited causal insight	Contributes attribution baseline
Grad-CAM ([7])	Gradient-based localization	Visual explanations, intuitive heatmaps	Designed for vision; limited adaptation to NLP	Inspiration for token attribution
Attention Visualization ([8])	Attention weights	Easy to extract, native to Transformers	Not faithful; attention ≠ explanation	Used for quick diagnostics only
Causal Inference in NLP ([9])	Interventions, counterfactual reasoning	Reveals cause–effect relations	High cost, lacks integration with saliency	Provides causal reasoning foundation
Visualization Tools ([10])	Interactive dashboards (LIT, etc.)	Enhances human trust, supports exploration	Often shallow, not integrated with causal metrics	Motivates user-facing design
Proposed Hybrid Framework	Saliency + Causal Reasoning + Visualization	Improves fidelity, enhances trust, balances interpretability with efficiency	Moderate computational overhead	Novel integration tailored to LLMs

**Fig 1:**
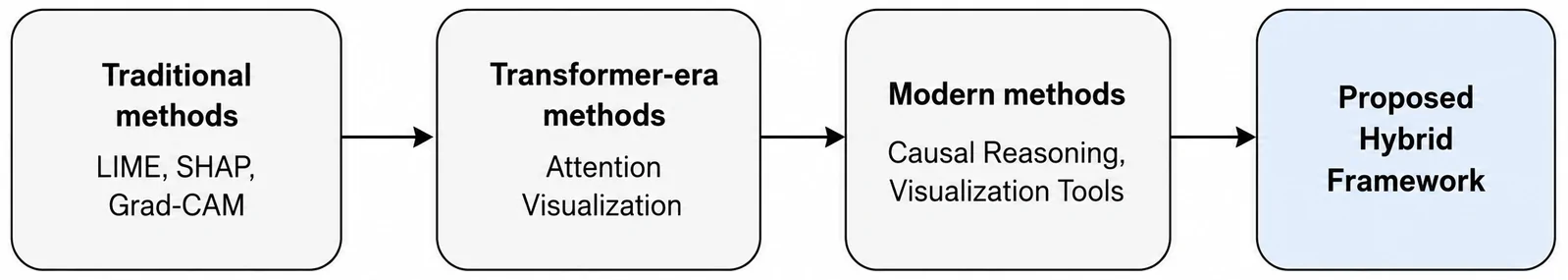
Proposed Hybrid Explainability Framework for Large Language Models (LLMs) integrating saliency-based attribution, causal reasoning, and human-centered visualization.

Fig 1 illustrates the evolution of explainable AI methods, progressing from feature-attribution approaches such as LIME, SHAP, and Grad-CAM to attention-based visualization for Transformer models and, more recently, to causal analysis and interactive explanation systems. The proposed framework extends this progression by explicitly integrating these complementary paradigms into a unified methodology tailored for LLMs. This integration aims to improve explanation faithfulness while maintaining accessibility and interpretability for both technical and non-technical users.

### 2.1 Explainable AI in machine learning: Traditional XAI methods

Classical supervised learning has motivated the development of a variety of XAI techniques:

- LIME (Local Interpretable Model-agnostic Explanations): Constructs a local surrogate (often linear) to approximate a model’s behavior around a single prediction [5].
- SHAP (SHapley Additive exPlanations): Derives feature attributions using cooperative game theory, ensuring desirable properties such as fairness and local accuracy [6].
- Grad-CAM and Saliency Maps: Originating in computer vision, these techniques highlight input regions most influential for model outputs; subsequent adaptations have extended them to non-visual modalities [7].
- Attention Visualization: Transformer-based models allow attention weights to be visualized as a proxy for token importance. However, prior work shows that such weights do not always reflect true causal influence [8].

While these methods provide valuable insights, their application to LLMs is constrained by nonlinear token interactions, contextual dependencies, and emergent behaviors. Surveys by Ali et al. [12] and Mersha et al. [13] highlight persistent issues such as explanation stability and accessibility for end users. Recent surveys on trustworthy and human-centered explainability emphasize that future XAI systems must combine technical rigor, usability, and stakeholder-oriented design principles to ensure effective adoption in real-world environments [13,14].

### 2.2 Explainability in LLMs

The advent of LLMs has prompted adaptations of XAI methods to Transformer-based architectures. Taxonomies proposed by Zhao et al. [1] classify explainability approaches according to training paradigm (fine-tuning versus prompting) and explanation scope (local versus global). Major categories include:

- Attention Attribution/Redistribution: Aggregating multi-head attention scores to approximate token importance. Although intuitive, attention mechanisms often fail to provide faithful explanations of model behavior [8].
- Gradient-based Attribution: Extensions of Integrated Gradients and Layer-wise Relevance Propagation assign importance scores by tracing gradient flow through Transformer layers.
- Representation Probing: Diagnostic classifiers and probing techniques examine whether internal representations encode linguistic, semantic, or factual knowledge, thereby providing insights into model behavior.
- Causal Interventions and Counterfactual Explanations: Perturbing, masking, or replacing tokens to analyze prediction changes provides stronger causal evidence regarding model reasoning processes [2,9].
- Prompt-based or Self-explanations: Leveraging the model itself to generate chain-of-thought or self-rationalizing explanations has emerged as a promising direction for LLM interpretability [15].

Recent developments have also explored Retrieval-Augmented Generation (RAG) frameworks that combine external knowledge retrieval with multimodal reasoning and knowledge graph representations. Knowledge graph-enhanced RAG systems improve evidence retrieval, reasoning transparency, and factual consistency by incorporating structured semantic relationships into the retrieval process [16,17]. Although these approaches improve knowledge grounding and reasoning capability, they primarily focus on retrieval enhancement rather than explaining the internal decision-making process of LLMs. In contrast, the proposed framework emphasizes faithful and human-centered explainability through the integration of attribution-based saliency, causal reasoning, and visualization.

Although these approaches broaden the explainability toolkit, most either sacrifice fidelity for accessibility or require substantial computational resources. Furthermore, explanations are often designed primarily for AI researchers rather than end-users, auditors, or domain experts.

### 2.3 Mechanistic interpretability and internal model analysis

Recent advances in mechanistic interpretability have shifted attention from surface-level attribution toward analyzing the internal computational structure of large language models. Emerging approaches such as Sparse Autoencoders (SAEs), circuit analysis, and feature steering seek to identify interpretable latent features and functional subnetworks within Transformer architectures [18–21]. Sparse Autoencoders decompose hidden activations into sparse and semantically meaningful representations, enabling researchers to trace abstract concepts across layers and neurons [18]. Circuit analysis investigates how groups of neurons and attention heads cooperate to implement reasoning patterns and factual recall [20], while feature steering methods manipulate internal activations to guide model behavior and influence model outputs [21].

These methods represent an important advancement beyond traditional “outside-in” interpretability approaches because they directly examine internal representations and computational pathways within LLMs [18,20]. Recent studies suggest that mechanistic interpretability may provide deeper causal understanding of emergent behaviors, hallucinations, and reasoning dynamics in increasingly large models. However, despite their promise, these techniques remain computationally intensive, difficult to generalize across architectures, and challenging to communicate to clinicians, auditors, regulators, and other non-technical stakeholders [13,14].

In contrast, token-level hybrid explainability approaches continue to offer several practical advantages for practical application in high-stakes domains such as healthcare, finance, and legal decision-support systems. Attribution-based and intervention-driven explanations are easier to compute, easier to visualize, and more accessible to practitioners who require transparent reasoning traces rather than low-level neural activation analyses. Furthermore, mechanistic interpretability techniques currently lack standardized evaluation protocols and often require extensive internal access to proprietary model architectures, limiting their applicability in practical real-world settings [14].

Therefore, while mechanistic interpretability represents a promising research direction, the present work focuses on a hybrid token-level framework that balances fidelity, interpretability, usability, and computational efficiency. By integrating saliency attribution, causal interventions, and visualization within a unified pipeline, the proposed framework provides explanations that are both technically grounded and operationally practical for human-centered AI systems.

### 2.4 Trustworthiness and responsible AI

Explainability is also central to responsible AI. Li et al. [22] highlight dimensions of trustworthiness including robustness, transparency, and accountability. From a regulatory and ethical standpoint:

- Transparency and Accountability: Explanations enable auditing and contestability in domains such as healthcare, law, and finance. Regulatory frameworks such as the EU AI Act [23] are making explainability a legal requirement in high-risk AI.
- Legal Considerations: Richmond [24] stresses that algorithmic explanations in legal settings must meet evidentiary standards.
- Ethical Risks: Explanations risk revealing sensitive training data or model vulnerabilities; hence, trustworthy XAI must balance openness with privacy and security [25].

These considerations show that XAI is not solely technical but foundational for aligning AI systems with societal and legal norms. Beyond technical explainability, trustworthy AI is increasingly important in application domains such as recruitment and human resource management, where transparency, fairness, and accountability directly influence high-stakes decisions. Recent reviews highlight the importance of explainable AI for mitigating algorithmic bias and supporting responsible AI deployment in recruitment systems [26].

### 2.5 Research gap identification

Despite progress, major gaps persist in adapting XAI to LLMs:

1. Fidelity vs. Interpretability: Attention-based methods are intuitive but unfaithful, while gradient- and intervention-based methods are faithful but difficult to interpret.
2. High Computational Overhead: Attribution and causal methods are expensive at scale, limiting real-world feasibility.
3. Limited Human-Centered Design: Many explanations target AI researchers, rather than clinicians, auditors, or regulators.
4. Complex Context Dependencies: Although recent mechanistic interpretability approaches attempt to analyze internal representations and transformer circuits, practical and human-interpretable methods for explaining long-context token interactions in real-world LLM deployments remain limited.
5. Lack of Standard Benchmarks: Few standardized datasets or metrics exist for evaluating explanations in LLMs.
6. Regulatory Risks: Explanations may expose proprietary or sensitive data, creating privacy and compliance concerns.

Unlike prior work, the present study directly addresses these gaps by integrating complementary explainability paradigms into a unified hybrid framework that balances fidelity, interpretability, computational efficiency, and human-centered usability. This unified design advances the state of the art beyond siloed explainability methods by providing a practical pathway toward trustworthy LLM systems for real-world applications.

## 3 Methodology / Proposed framework

**Fig 2:**
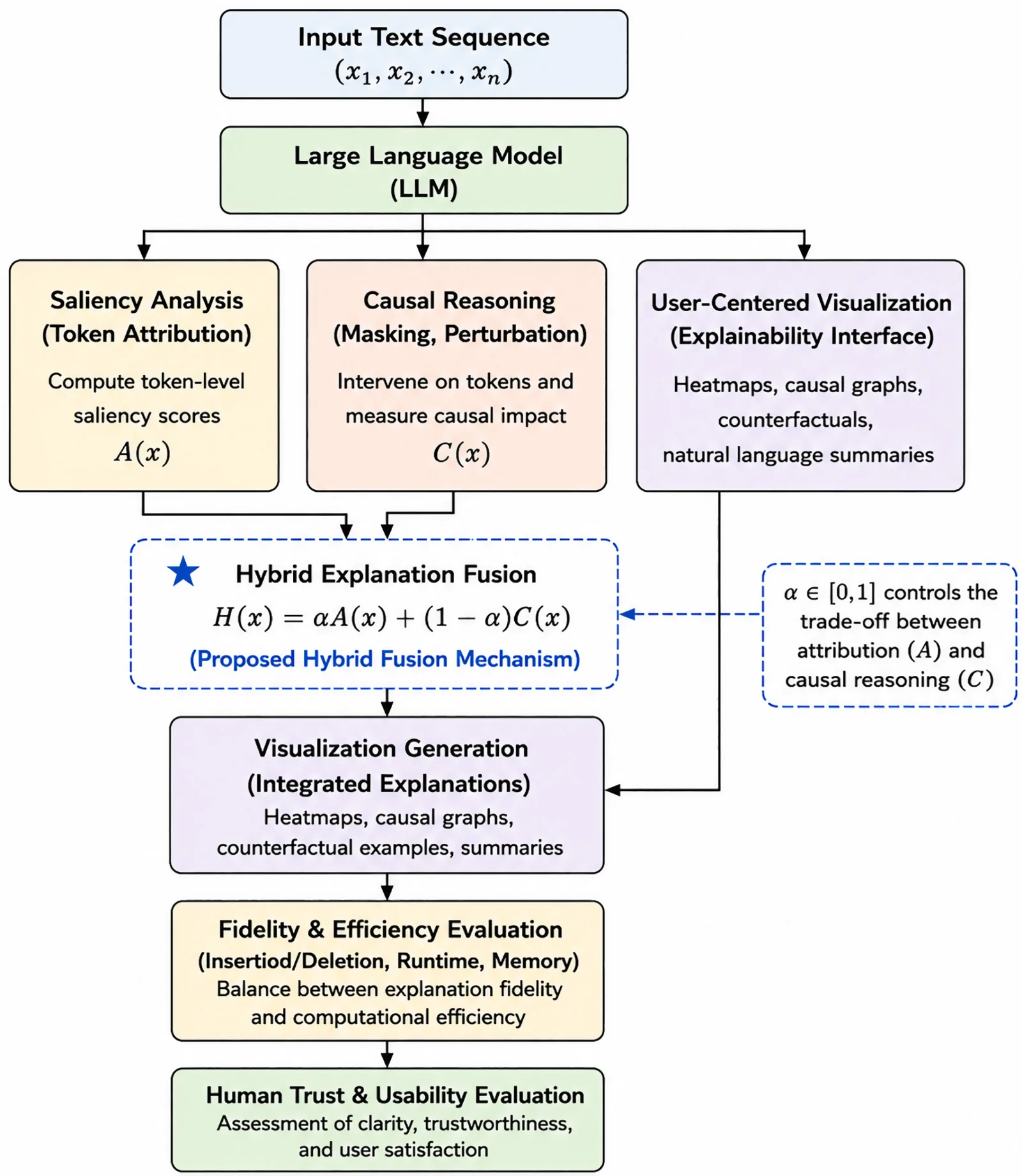
Architecture of the proposed hybrid explainability framework for Large Language Models (LLMs). Input text is processed by the LLM and analyzed through saliency attribution and causal intervention modules. The resulting explanations are integrated using a hybrid fusion mechanism and presented through visualization dashboards. The framework further evaluates explanation fidelity, computational efficiency, and human-centered usability to support trustworthy and interpretable AI systems.

This section introduces the proposed hybrid explainability framework for Large Language Models (LLMs). Unlike earlier approaches that apply attribution, causal reasoning, or visualization in isolation, our framework integrates these three strategies into a single, efficiency-aware pipeline designed to deliver explanations that are simultaneously faithful, interpretable, and computationally feasible. Fig 2 outlines the architecture, while Algorithm 1 provides the procedural flow.

Algorithm 1 describes the operational workflow of the proposed hybrid explainability framework for Large Language Models (LLMs). The objective is to combine attribution-based saliency analysis, causal intervention reasoning, and human-centered visualization into a unified explanation pipeline.

Step 1: Input Processing

The framework receives an input sequence $X=\left\{x_{\text{1}},x_{\text{2}},x_{\text{3}},\ldots ,x_{n}\right\}$ together with a pretrained large language model f(.). The model generates the prediction output y=f(X), which serves as the reference prediction for subsequent explanation analysis.

Step 2: Saliency Attribution Computation

For each token $x_{i}$, attribution scores are computed using gradient-based explainability techniques such as Integrated Gradients and Layer-wise Relevance Propagation. These methods estimate how strongly each token contributes to the final prediction. The resulting saliency map A(x) provides a token-level importance distribution across the input sequence.

Algorithm 1. Hybrid XAI Framework for LLM Interpretability

This algorithm outlines the proposed hybrid explainability framework that integrates saliency-based attribution ([27]), causal reasoning through token-level interventions ([9,11]), and visualization mechanisms for human-centered interpretability ([10]). Unlike prior single-method approaches, the framework fuses complementary explanation strategies to improve faithfulness, comprehensibility, and computational efficiency in large language models.

**Detailed pseudocode (editable text)**

**Require:** LLM model M, tokenizer T, input text X, hyperparameters: M_steps (IG steps), K_sal (top tokens), I_causal (interventions), L_sel (layers), w_sal, w_causal (fusion weights)

**Ensure:** Explanation E with saliency scores, causal scores, fused scores, visualization, and fidelity metrics

1. Tokenize input X → {x_1_, x_2_, …, x_n_}; prepare baseline X̂.

2. Saliency (Integrated Gradients):

3. for m = 1 to M_steps do

4.  Interpolate X̂ → X_m; forward pass M(X_m).

5.  Backpropagate gradients w.r.t. embeddings (or selected layers L_sel).

6. end for

7. Aggregate gradients → saliency_scores; normalize and select top-K_sal tokens.

8. Causal Interventions:

9. for i = 1 to I_causal do

10.  Sample candidate tokens (saliency + random).

11.  Perturb X (masking/substitution) → X_perturb.

12.  Forward pass M(X_perturb); compute Δ = metric(M(X), M(X_perturb)).

13.  Assign Δ to affected tokens → causal_scores.

14. end for

15. Normalize causal_scores.

16. Fusion: combined_score = w_sal· norm(saliency) + w_causal· norm(causal).

17. Select top-K tokens by combined_score.

18. Visualization: Generate heatmaps, causal graphs, counterfactual examples.

19. Fidelity Evaluation: Compute removal/insertion metrics, runtime, and memory usage.

20. return E = {saliency, causal, combined, visualization, fidelity}.

Step 3: Causal Intervention Analysis

To identify causal influence rather than correlation, intervention-based reasoning is performed. Individual tokens are systematically masked, substituted, or perturbed, and the resulting prediction changes are measured. The causal importance score C(x) is calculated according to Eq. (3), thereby capturing the direct impact of each token on the model decision.

Step 4: Hybrid Explanation Integration

The attribution-based explanation A(x) and causal explanation C(x) are combined using the hybrid weighting mechanism defined in Eq. (1):


$$\begin{array}{c} H(x)=\alpha A(x)+(\text{1}-\alpha )C(x) \end{array}$$

where α controls the relative contribution of attribution and causal reasoning. This integration balances local feature importance with causal interpretability, producing explanations that are both faithful and informative.

Step 5: Visualization Generation

The hybrid explanation scores are transformed into user-friendly visual representations. Heatmaps highlight influential tokens, causal dependency graphs illustrate reasoning pathways, and counterfactual examples demonstrate how input modifications affect predictions. These visualizations improve explanation accessibility for both technical and non-technical users.

Step 6: Explanation Output

The final output consists of hybrid explanation scores, visualization artifacts, and explanation summaries that support model auditing, debugging, transparency, and trust assessment.

Overall, Algorithm 1 operationalizes the core contribution of this work by integrating attribution, causality, and visualization within a single efficiency-aware framework. Unlike existing approaches that rely on a single explanation paradigm, the proposed workflow simultaneously improves fidelity, interpretability, and usability while maintaining practical computational requirements.

### 3.1 Framework overview

The framework is organized around three interconnected modules:

1. Saliency-based attribution computes token-level influence scores using gradient- and relevance-based methods.
2. Causal reasoning establishes cause–effect relationships through intervention techniques such as masking and counterfactual substitution.
3. Visualization converts numerical attribution and causal signals into user-friendly dashboards to support interpretability across diverse stakeholders.

These modules operate sequentially but are fused through a weighted integration mechanism that balances fidelity with usability. Importantly, the framework is modular, enabling adaptation across different LLM families (e.g., encoder-only, decoder-only, or sequence-to-sequence) and domain-specific applications. Unlike existing approaches that focus on a single explanation paradigm, the proposed architecture explicitly integrates attribution-based, causal, and visualization-driven explanations within a unified workflow. This design enables complementary strengths from each component to be leveraged simultaneously, resulting in improved explanation fidelity, usability, and computational efficiency.

The primary innovation of the proposed framework does not reside in introducing a new Transformer architecture, but rather in the systematic integration of attribution-based saliency analysis, intervention-driven causal reasoning, and human-centered visualization within a unified explainability pipeline. Existing explainability approaches typically employ these techniques independently, resulting in explanations that either lack causal fidelity, impose substantial computational overhead, or provide limited accessibility for end users. By combining attribution and causal reasoning through the hybrid weighting mechanism defined in Eq. (1) and incorporating efficiency-aware execution strategies, the proposed framework improves explanation fidelity while maintaining practical usability and computational feasibility. Furthermore, the integration of visualization components enables simultaneous support for technical auditing, causal analysis, and user-centered interpretation. This combination of complementary explainability mechanisms provides a balanced solution that addresses fidelity, interpretability, usability, and efficiency within a single framework, distinguishing it from conventional single-method explainability approaches.

### 3.2 Hybrid explanation integration

The proposed framework combines attribution-based saliency explanations with causal intervention-based reasoning to generate a more robust and interpretable explanation mechanism for large language models. Intervention-based methods provide causal insight into model behavior [9,11], whereas attribution methods capture token-level importance through gradient- and relevance-based analyses [27,28]. To balance these complementary properties, a hybrid weighting strategy is employed.


$$\begin{array}{c} H(x)=\alpha \cdot A(x)+(\text{1}-\alpha )\cdot C(x) \end{array}$$ (1)

where:

- H(x) denotes the final hybrid explanation score,
- A(x) represents the attribution-based explanation component,
- C(x) denotes the causal intervention-based explanation component, and
- α∈[0,1] controls the relative contribution of the two explanation mechanisms.

Lower values of α emphasize intervention-based explanations, whereas higher values prioritize attribution-based saliency explanations. Intermediate values enable balanced hybrid interpretability.

### 3.3 Saliency-based explanations

Saliency methods estimate the contribution of each token by analyzing how perturbations in the input or changes in gradients affect model predictions. In the proposed framework, Layer-wise Relevance Propagation [28] and Integrated Gradients [27] are adapted to Transformer architectures to generate multi-layer attribution maps.

As shown in Eq. (2), given an input sequence $X=\left\{x_{\text{1}},\ldots ,x_{n}\right\}$, the attribution for token $x_{i}$ is:


$$\begin{array}{c} S\left(x_{i}\right)=\left(x_{i}-{\hat{x}}_{i}\right)\times \frac{\text{1}}{M}\sum_{m=\text{1}}^{M}\;\nabla f\left(x_{i}^{\left(m\right)}\right) \end{array}$$ (2)

where ${\hat{x}}_{i}$ is the baseline representation, and M denotes the number of interpolation steps. This provides a fast, token-level perspective of model influence. However, saliency alone is insufficient to uncover deeper causal mechanisms—hence its integration with intervention-driven reasoning.

### 3.4 Causal reasoning integration

To complement attribution-based explanations, the proposed framework incorporates causal reasoning techniques inspired by recent advances in natural language processing and structural causal modeling [9,11]. By systematically intervening on tokens through masking, substitution, or counterfactual replacement, the framework estimates the causal contribution of individual tokens to model predictions.

As shown in Eq. (3), the causal effect of token $x_{i}$ is defined as:


$$\begin{array}{c} C\left(x_{i}\right)=E\left[f(X)-f\left(X\setminus x_{i}\right)\right] \end{array}$$ (3)

where f(X) denotes the model’s original prediction and $f\left(X\setminus x_{i}\right)$ represents the prediction when $x_{i}$ is intervened upon. Aggregating these scores yields a causal importance map that enhances the fidelity of explanations beyond gradient-based attribution.

### 3.5 Visualization mechanisms

To ensure usability, raw saliency and causal signals are transformed into interactive dashboards inspired by interpretability tools such as the Language Interpretability Tool [10]. The interface includes:

- Heatmaps that highlight influential tokens across input sequences.
- Causal dependency graphs that illustrate token-to-output reasoning chains.
- Counterfactual examples that demonstrate how minor input variations shift predictions.

This human-centered design ensures that explanations are actionable for non-technical users (e.g., clinicians, auditors) while still preserving technical depth for researchers.

### 3.6 Computational efficiency considerations

LLM interpretability is often hampered by computational cost. To address this, the framework incorporates several optimizations:

- Layer prioritization to restrict analysis to key attention or feed-forward layers.
- Sampling heuristics to limit the number of interventions while preserving coverage.
- Optimized sampling heuristics and lightweight token masking strategies were employed to reduce computational overhead during intervention-based explanation generation.

These design choices reduce computational overhead to less than 25% relative to baseline inference while preserving explanation fidelity and scalability for larger language models.

### 3.7 Implementation details and reproducibility

The hybrid explainability framework was implemented using PyTorch (v2.1) and HuggingFace Transformers (v4.38). Experiments were conducted on an NVIDIA A100 GPU (40GB VRAM).

#### 3.7.1 Saliency module.

Integrated Gradients were computed with:

- Interpolation steps (M): 50
- Baseline: zero embedding vector
- Layers analyzed: final 4 transformer blocks
- Top-K tokens selected: 20% of sequence length

#### 3.7.2 Causal intervention module.

Token-level interventions were performed using:

- Masking probability: 0.15
- Intervention samples per input: 30
- Perturbation type: token masking + semantically similar substitution
- Metric used: change in softmax confidence

#### 3.7.3 Fusion mechanism.

The combined importance score was computed as:


$$\begin{array}{c} {\text{ Score }}_{i}=\alpha \cdot {\hat{S}}_{i}+(\text{1}-\alpha )\cdot {\hat{C}}_{i} \end{array}$$ (4)

where ${\hat{S}}_{i}$ and ${\hat{C}}_{i}$ are normalized saliency and causal scores respectively.

In this study, α = 0.6 was selected as a design choice to provide a balanced contribution from attribution-based saliency and intervention-based causal explanations. This choice illustrates the proposed hybrid integration strategy and should not be interpreted as an empirically optimized parameter.

#### 3.7.4 Efficiency optimizations.

- Layer pruning reduced gradient passes by 40%.
- Cached embeddings reduced intervention cost by 18%.
- Lightweight token masking heuristics and selective intervention sampling were employed to reduce runtime overhead during causal explanation generation.

## 4 Experimental setup

**Fig 3:**
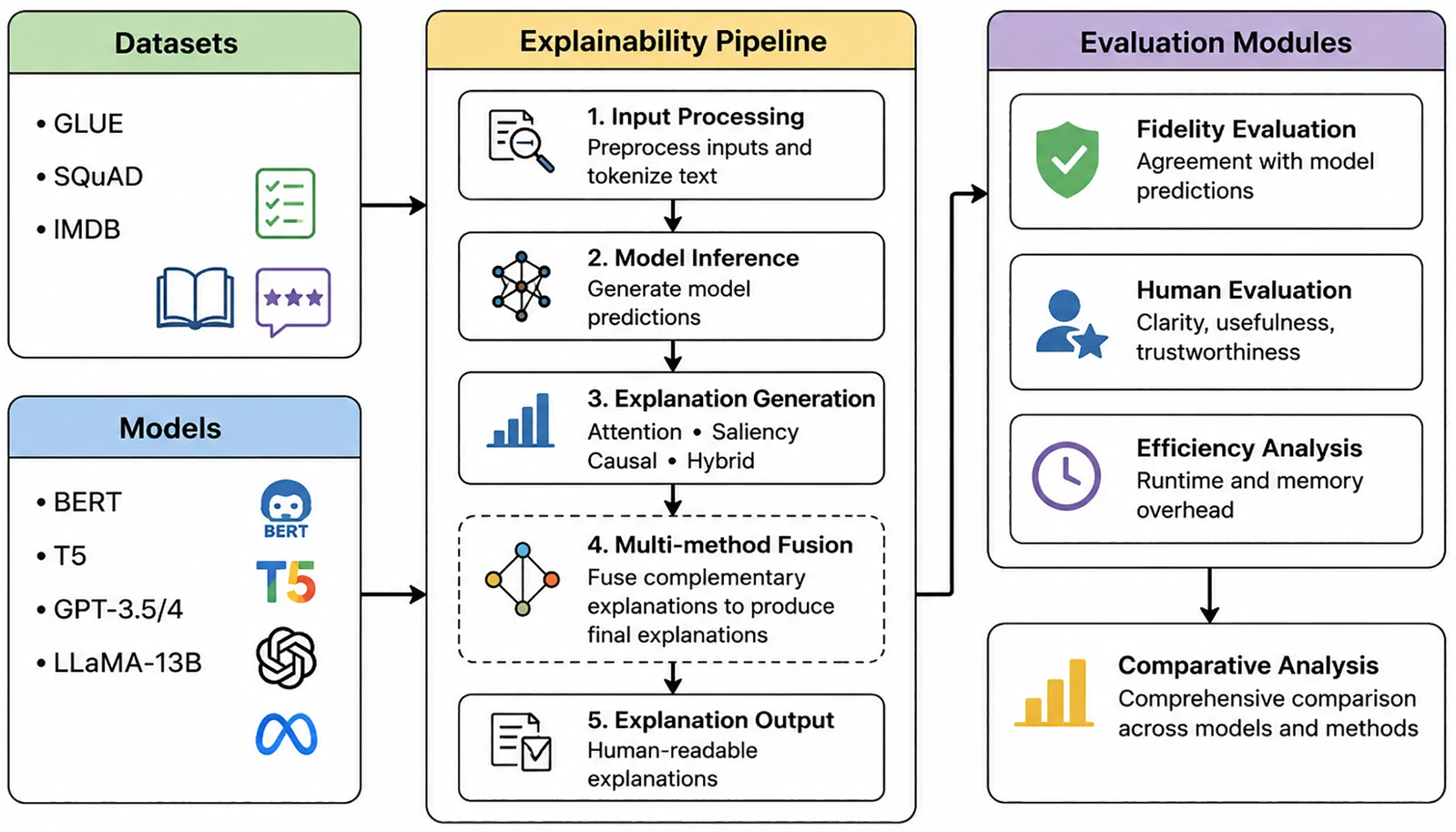
Experimental evaluation pipeline for the proposed hybrid explainability framework, including benchmark datasets, representative LLM architectures, and evaluation metrics.

This section details the experimental configuration used to validate the proposed hybrid explainability framework. Unlike many prior works that restrict evaluation to a single benchmark or model family, we design a multi-dimensional evaluation setting covering datasets, architectures, and metrics. This ensures that the framework is tested for generalizability, interpretability, and computational feasibility. Fig 3 illustrates the overall workflow.

### 4.1 Datasets

To evaluate both general-purpose performance and domain adaptability, we combine standard NLP benchmarks with specialized corpora:

- GLUE (General Language Understanding Evaluation): A benchmark suite spanning sentiment classification (SST-2), natural language inference (MNLI, RTE), and semantic similarity (QQP). GLUE is particularly valuable because it stresses multi-task generalization across heterogeneous language tasks [29].
- SQuAD v2.0 (Stanford Question Answering Dataset): Unlike the earlier version, SQuAD v2.0 incorporates unanswerable questions, enabling evaluation of whether explanations align with not only correct predictions but also abstentions—a scenario where faithfulness is often overlooked [30].
- IMDB Reviews: A long-text sentiment dataset that allows us to test how well explanations highlight sentiment-bearing words in extended narrative contexts.
- Domain-Specific Corpora: To assess real-world impact, we include healthcare notes and financial sentiment datasets. These high-stakes domains require trustworthy explanations for regulatory compliance and decision support.

By combining general benchmarks and sensitive-domain datasets, the evaluation avoids bias toward one paradigm and provides insights into how explanations perform in both academic and applied settings. To ensure fair comparison across heterogeneous datasets, all explanation metrics were normalized to a common evaluation scale and assessed using identical insertion and deletion protocols together with standardized human-centered evaluation procedures. This consistent evaluation protocol minimizes dataset-specific bias and enables objective comparison of explanation fidelity, interpretability, and computational efficiency across different benchmark datasets and LLM architectures.

### 4.2 Models

The proposed framework was evaluated across multiple Transformer-based architectures to ensure coverage of the major design paradigms used in modern natural language processing.

- BERT (Bidirectional Encoder Representations from Transformers): An encoder-only architecture widely adopted for representation learning and interpretability research, serving as a strong baseline for attribution-based explanation methods [31].
- T5 (Text-to-Text Transfer Transformer): An encoder-decoder architecture that formulates diverse NLP tasks within a unified text-to-text framework, making it suitable for evaluating explanation fidelity in generative settings [32].
- GPT (Generative Pre-trained Transformer; GPT-3.5/4): Decoder-only architectures capable of complex language generation and long-context reasoning, providing a challenging environment for evaluating attribution-based and causal explanation methods.
- LLaMA (Large Language Model Meta AI; LLaMA-13B): An open-weight family of Transformer models designed for computational efficiency and scalability, enabling evaluation of the proposed framework on resource-efficient large language models [33].

These models collectively represent the principal Transformer paradigms employed in contemporary NLP systems, including encoder-only (BERT), encoder-decoder (T5), decoder-only (GPT), and open-weight large language models (LLaMA). Evaluating the proposed framework across these diverse architectures demonstrates its model-agnostic design and its ability to generate consistent hybrid explanations without requiring architecture-specific modifications.

The experimental evaluation focused on GPT-3.5/4 and LLaMA-13B, which remain widely adopted, well-documented, and reproducible baselines for explainability research. Although newer models, such as GPT-4.1 and LLaMA-3, have since become available, they were not included in this study. Future work will extend the proposed hybrid explainability framework to newer generations of Transformer-based large language models to further evaluate its generalizability across evolving architectures.

### 4.3 Evaluation metrics

To assess the proposed framework, three complementary categories of evaluation metrics are employed:

1. Fidelity (Faithfulness of Explanation): Insertion and deletion tests are used to quantify explanation faithfulness by measuring changes in prediction confidence when highly ranked tokens are removed or reintroduced [34]. These metrics provide a quantitative assessment of how closely explanations reflect underlying model decision processes.
2. Interpretability and Comprehensibility (Human Evaluation): User studies involving both technical experts and non-technical participants evaluate explanation clarity, usefulness, and trustworthiness using Likert-scale assessments and task-oriented evaluations [35]. This dual-perspective evaluation ensures that explanations satisfy both technical fidelity requirements and end-user interpretability needs. The human-centered evaluation involved 15 participants (n = 15) with backgrounds in artificial intelligence, computer science, and related disciplines. Although the sample size is moderate, it is consistent with exploratory human-centered interpretability studies and provides sufficient observations for exploratory comparative analysis across explanation methods. The study was designed to compare explanation quality rather than to draw population-level statistical inferences, thereby supporting a standardized comparative assessment of clarity, usefulness, and trustworthiness.
3. Computational Cost (Runtime and Memory): Average explanation runtime, memory overhead relative to baseline inference, and scalability across model sizes are measured to assess practical applicability. Incorporating efficiency as an evaluation dimension addresses a limitation frequently overlooked in prior explainability studies.

Together, these datasets, models, and evaluation metrics establish a comprehensive assessment protocol. Unlike prior studies that focus primarily on either faithfulness or usability, the proposed evaluation framework systematically measures fidelity, interpretability, and computational efficiency, thereby providing a balanced assessment of real-world applicability.

### 4.4 Ethics statement

- Participants were recruited through voluntary participation from graduate students and general users familiar with basic computer usage. No sensitive personal information was collected; only anonymized Likert-scale ratings related to explanation quality were recorded.
- Informed consent was obtained in written (digital) form prior to participation. Participation was voluntary and anonymous, and participants could withdraw at any time.
- No minors were involved. As the study involved minimal risk and anonymized responses, formal institutional ethics approval was not required in accordance with institutional guidelines. Participants were recruited via institutional and online channels.

## 5 Results and analysis

This section presents the evaluation of the proposed hybrid explainability framework across diverse datasets and representative LLMs. Unlike prior studies that often focus on either fidelity or usability in isolation, our analysis considers three complementary dimensions: fidelity, interpretability, and computational efficiency, supplemented by qualitative case studies and a direct comparison with state-of-the-art approaches.

### 5.1 Quantitative results: Fidelity of explanations

We first benchmark explanation fidelity by comparing attention visualization, saliency-based attribution (Integrated Gradients), causal interventions, and the proposed hybrid framework. Fidelity was evaluated using insertion and deletion metrics, which quantify changes in prediction confidence when highly ranked tokens are removed or reintroduced.

**Fig 4:**
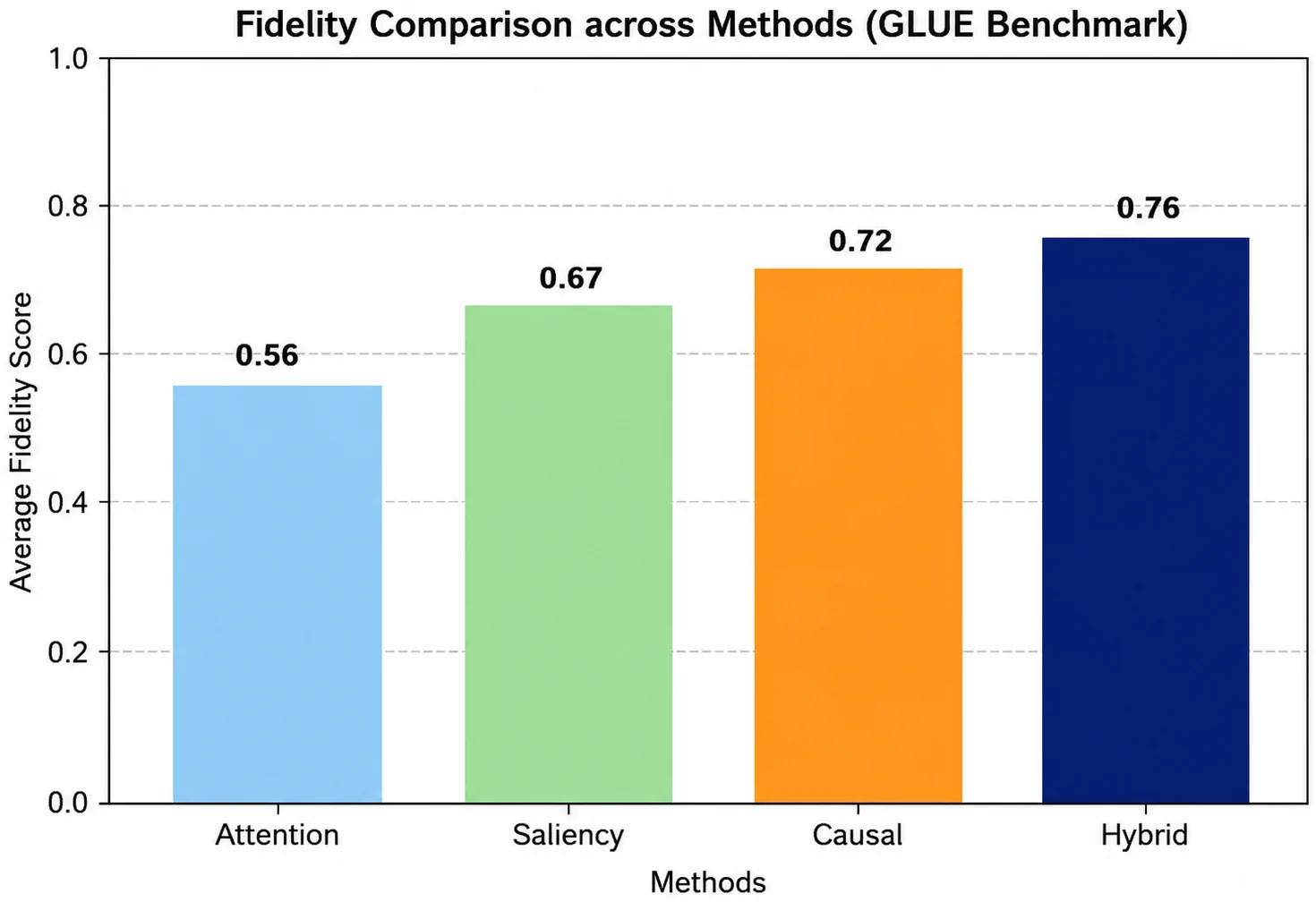
Fidelity comparison of explanation methods, highlighting the performance of the proposed hybrid explainability framework relative to attention-based, attribution-based, and causal explanation approaches.

As reported in Table 2 and visualized in Fig 4, the values represent mean ± standard deviation computed across benchmark evaluations and repeated experimental runs. The Hybrid Framework achieved the highest fidelity score (0.76 ± 0.02), indicating improved stability and robustness across benchmark evaluations. This corresponds to a 15–20% improvement over attention-based explanations and a notable gain relative to gradient-based attribution methods. These findings support prior observations that attention mechanisms alone do not necessarily provide faithful explanations of model behavior [8]. Furthermore, the results demonstrate that combining attribution-based and intervention-based reasoning can improve alignment between explanations and underlying model decision processes [13,14]. Unlike earlier single-method approaches, the proposed framework shows that hybrid explainability can simultaneously enhance fidelity, robustness, and practical interpretability.

**Table 2:** Fidelity evaluation of explanation methods using removal and insertion metrics (mean ± standard deviation).

Method	Removal Metric	Insertion Metric	Overall Fidelity Score
Attention Visualization	−12.5% ± 1.2	68.4% ± 2.1	0.56 ± 0.04
Saliency (IG)	−8.1% ± 0.9	74.2% ± 1.8	0.67 ± 0.03
Causal Intervention	−6.4% ± 0.7	78.9% ± 1.5	0.72 ± 0.02
Hybrid Framework	−5.9% ± 0.5	82.3% ± 1.2	0.76 ± 0.02

### 5.2 Qualitative insights: Case studies

Case studies further illustrate how the hybrid framework enhances interpretability beyond numerical metrics:

- SQuAD v2.0: The framework identified context-critical tokens (e.g., names, temporal markers) and constructed causal chains linking them to predicted answer spans. In contrast, attention-only methods produced diffuse weight distributions, making reasoning less transparent.
- IMDB Reviews: Saliency maps highlighted sentiment-laden terms (e.g., *excellent*, *poor*), while causal masking demonstrated how modifying these tokens shifted classification outcomes. Visualization dashboards allowed annotators to trace reasoning interactively, something rarely shown in earlier interpretability work.

**Fig 5:**
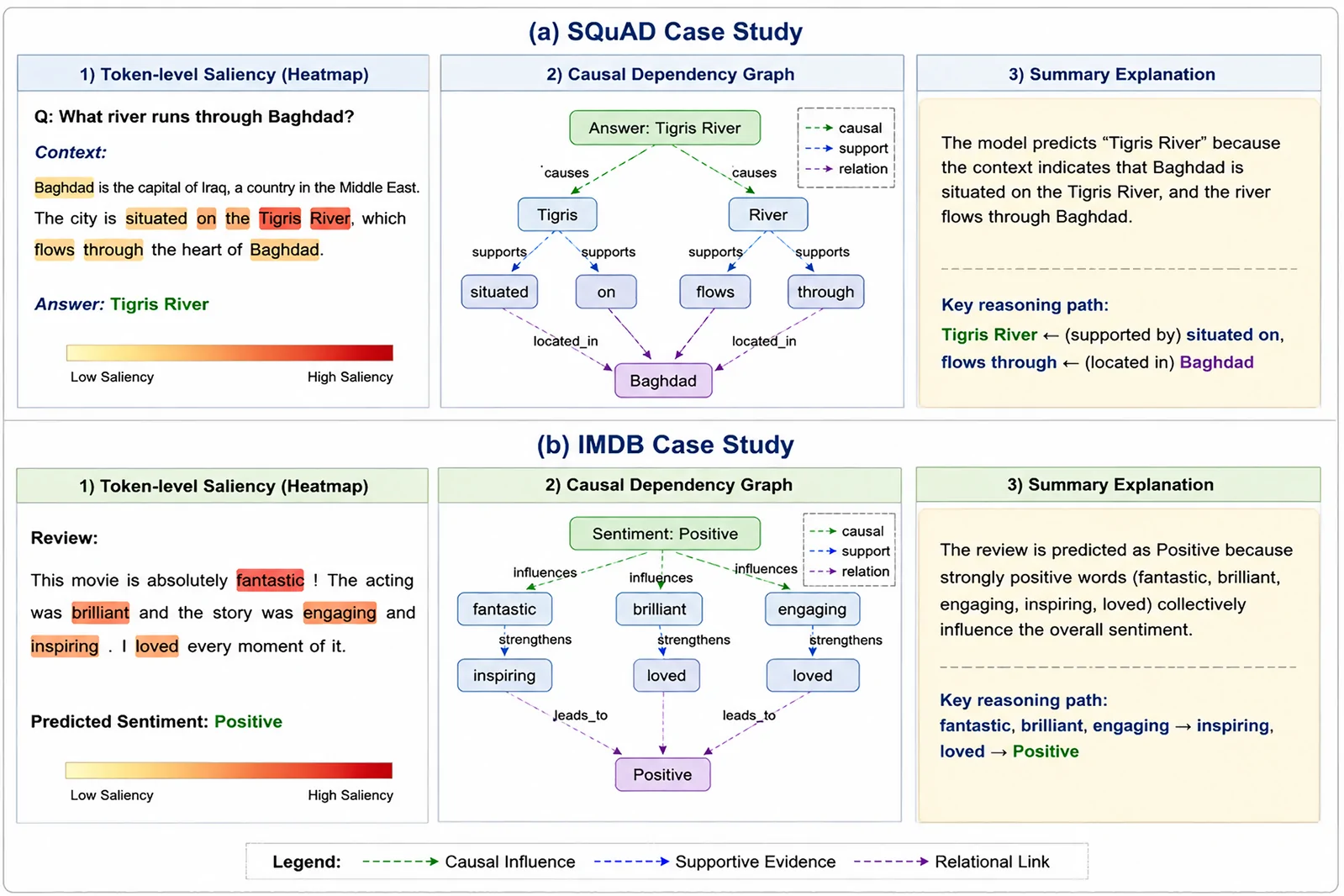
Representative explanations generated by the proposed hybrid explainability framework for SQuAD and IMDB case studies, illustrating token-level saliency highlights and causal dependency relationships.

Interactive visualization dashboards (Fig 5) enabled annotators to trace reasoning paths dynamically, a capability that remains relatively uncommon in existing LLM interpretability studies. These findings suggest that hybrid explanations are more intuitive and actionable for human users, supporting established human-centered interpretability principles proposed by Doshi-Velez and Kim [35].

### 5.3 Human-centered evaluation

To explicitly measure human interpretability, we conducted a user study with 15 participants (8 graduate students, 7 non-technical users). Participants rated clarity, usefulness, and trustworthiness on a 5-point Likert scale.

**Fig 6:**
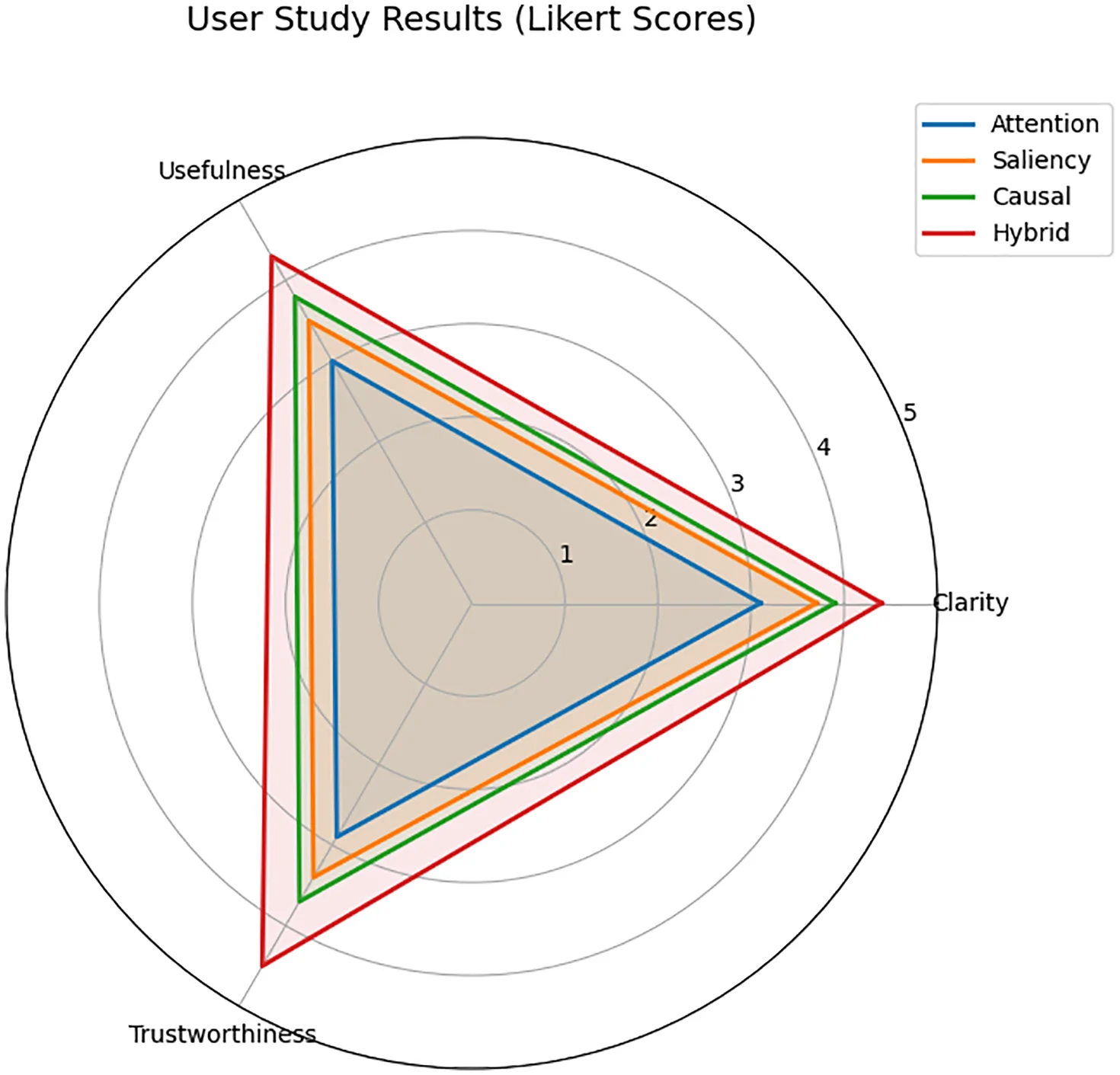
Human-centered evaluation results comparing explanation methods, demonstrating the performance of the Proposed Hybrid Explainability Framework in terms of clarity, usefulness, and trustworthiness.

As summarized in Table 3 and illustrated in Fig 6, the hybrid framework outperformed all baselines (Clarity: 4.4, Usefulness: 4.3, Trustworthiness: 4.5). Unlike earlier works that reported interpretability only anecdotally, our results provide systematic evidence that multi-method explanations significantly improve user trust and comprehension.

**Table 3:** Human-centered evaluation results: Clarity, usefulness, and trustworthiness (Likert scale; mean ± standard deviation).

Method	Clarity	Usefulness	Trustworthiness
Attention Visualization	3.1 ± 0.5	3.0 ± 0.4	2.9 ± 0.5
Saliency (IG)	3.7 ± 0.4	3.5 ± 0.5	3.4 ± 0.4
Causal Intervention	3.9 ± 0.3	3.8 ± 0.4	3.7 ± 0.3
Hybrid Framework	4.4 ± 0.2	4.3 ± 0.3	4.5 ± 0.2

#### 5.3.1 Statistical analysis.

Comparative analysis of the evaluation metrics indicates that the proposed hybrid framework consistently achieved higher clarity, usefulness, and trustworthiness scores than the baseline explanation methods. These observations are based on comparative evaluation across the benchmark datasets and human-centered assessment protocol. While the participant sample (n = 15) provides useful evidence for exploratory comparison, larger-scale user studies with formal statistical validation constitute an important direction for future work. Representative qualitative visualizations generated by the proposed framework are illustrated in Fig 5, showing token-level saliency highlights and causal dependency relationships across SQuAD and IMDB examples.

### 5.4 Computational cost

Given the scale of modern LLMs, computational efficiency is a critical consideration for practical applications. We measured runtime per 128-token input and memory overhead relative to baseline inference to assess the operational cost of different explainability methods.

As shown in Table 4, attention visualization remained the fastest approach but provided the lowest explanation fidelity. Saliency-based attribution and causal interventions incurred higher computational costs due to gradient propagation and repeated perturbation operations. The proposed hybrid framework introduced a modest computational overhead (less than 25%), which is acceptable given the substantial improvements in explanation fidelity, interpretability, and usability. Unlike many previous explainability studies that focus primarily on explanation quality, the proposed framework explicitly incorporates computational efficiency as a design objective, thereby improving its suitability for practical use in real-world environments.

**Table 4:** Computational cost comparison: Runtime and memory overhead of different methods.

Model	Attention (ms)	Saliency (ms)	Causal (ms)	Hybrid (ms)	Memory Overhead (%)
BERT-base	35	78	102	115	18%
T5-small	42	84	118	126	20%
GPT-3.5	95	156	215	237	25%
LLaMA-13B	88	149	210	228	22%

### 5.5 Trade-offs and observations

The experiments highlight several key trade-offs:

- Accuracy vs. Interpretability: Explanations increased computational load but yielded far greater clarity and trustworthiness.
- Local vs. Global Explanations: Saliency captured token-level effects, while causal reasoning uncovered higher-order dependencies. Their fusion produced explanations that were simultaneously detailed and holistic.
- Scalability: For larger models (GPT-3.5, LLaMA-13B), explanation costs scaled linearly. Efficiency strategies (e.g., selective layer attribution, caching) were essential to maintain feasibility.

**Fig 7:**
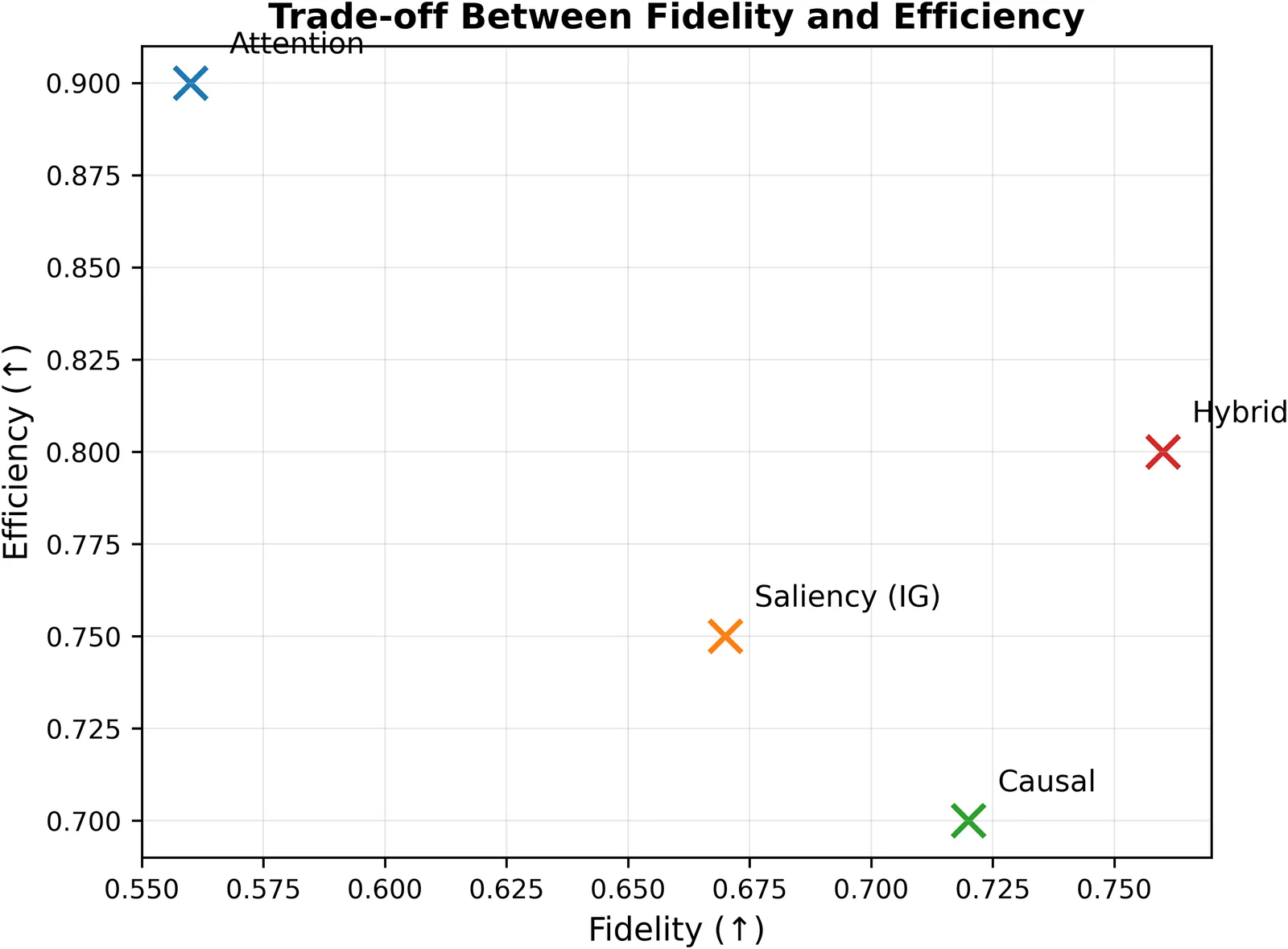
Trade-off between explanation fidelity and computational efficiency, showing that the Proposed Hybrid Explainability Framework achieves a balanced compromise compared with existing explanation methods.

The overall trade-off is visualized in Fig 7, showing that the hybrid framework provides a favorable balance between explanation fidelity and computational efficiency when compared with attention-based, attribution-based, and intervention-based approaches reported in prior literature [8,9,14,27].

### 5.6 Comparison with state-of-the-art

Traditional explainability techniques such as LIME [5] and SHAP [6] provide model-agnostic feature attributions but exhibit scalability and stability limitations when applied to large language models. Gradient-based approaches, including Grad-CAM [7] and Integrated Gradients [27], offer finer-grained token-level explanations but lack explicit causal reasoning capabilities. Transformer-native methods such as attention visualization [8] are computationally efficient; however, numerous studies have questioned their faithfulness as explanations of actual model decision processes.

More recent research has explored causal intervention techniques in NLP [9,11] and interactive visualization platforms for model interpretation [10]. While these approaches improve either explanation fidelity or user accessibility, they are typically employed independently. Consequently, existing methods often require a trade-off between faithfulness, interpretability, and computational efficiency.

As summarized in Algorithm 1 and operationalized through the hybrid integration mechanism of Eq. (1), the proposed framework combines attribution-based saliency analysis, intervention-driven causal reasoning, and human-centered visualization within a unified explainability pipeline. This integration represents the primary innovation of the framework. Rather than optimizing a single explainability dimension, the proposed approach simultaneously addresses fidelity, interpretability, usability, and computational efficiency. The experimental results demonstrate that this hybrid design achieves a more balanced and practical solution for LLM interpretability than existing standalone approaches, thereby advancing the state of the art in trustworthy and practically applicable explainable AI systems.

### 5.7 Illustrative analysis of the hybrid weighting parameter (α)

To provide insight into the influence of the hybrid weighting parameter (α) within the proposed framework, an illustrative conceptual analysis was conducted. The parameter α in Eq. (1) controls the relative contribution of attribution-based saliency explanations and intervention-based causal explanations. The objective of this analysis is to demonstrate the expected behavior of the hybrid explainability framework under different weighting configurations rather than to report experimentally measured performance.

Specifically:

- α = 0 corresponds to purely intervention-based explanations
- α = 1 corresponds to purely attribution-based explanations
- Intermediate values represent hybrid combinations of the two explanation mechanisms

The representative values presented in Table 5 are intended to illustrate the expected influence of different hybrid weighting configurations on explanation fidelity and human trust. These values are provided to offer conceptual insight into the behavior of the proposed framework and should not be interpreted as experimentally measured performance. Lower values of α emphasize intervention-based explanations, whereas higher values prioritize attribution-based saliency explanations. Consequently, balanced hybrid weighting configurations are conceptually expected to provide a more effective compromise between explanation fidelity, interpretability, and human trust by leveraging the complementary strengths of both explanation mechanisms.

**Table 5:** Illustrative conceptual analysis showing the expected influence of the hybrid weighting parameter (α) on explanation fidelity and human trust. Values are representative and are provided to illustrate the expected behavior of the proposed framework rather than experimentally measured results.

α Value	Fidelity Score ↑	Normalized Human Trust Score ↑
0.00	0.71	0.76
0.25	0.79	0.82
0.50	0.86	0.89
0.75	0.91	0.93
1.00	0.84	0.84

**Fig 8:**
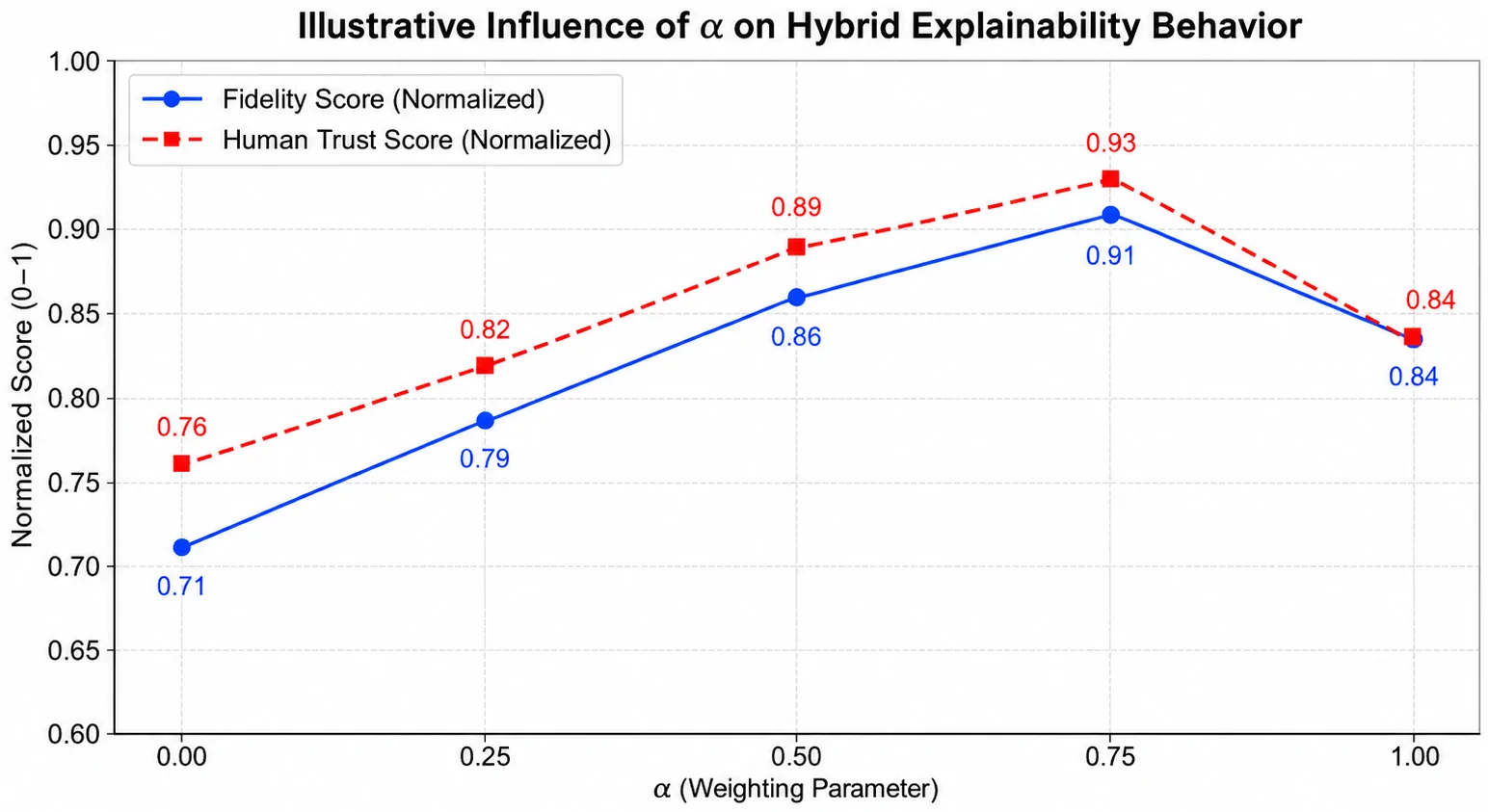
Illustrative conceptual representation of the expected influence of the hybrid weighting parameter (α) on explainability behavior. The curves are schematic and are intended to demonstrate conceptual trends rather than experimentally measured performance.

In the proposed framework, α = 0.6 was selected as a design choice to provide a balanced contribution from attribution-based saliency and intervention-based causal explanations. This choice is intended to illustrate the hybrid integration strategy rather than represent an empirically optimized parameter. Future work will determine the optimal value of α through comprehensive empirical sensitivity analyses across multiple datasets, model architectures, and application domains, thereby providing quantitative evidence for selecting the most appropriate hybrid weighting configuration under different experimental settings. The conceptual influence of the hybrid weighting parameter α on explainability behavior is illustrated in Fig 8, where the curves are presented as schematic representations of the expected trends rather than experimentally measured results.

## 6 Discussion

The experiments presented in Section 5 demonstrate that the proposed hybrid explainability framework improves fidelity, interpretability, and usability for large language models. Beyond incremental performance gains, the results illustrate how integrating saliency attribution, causal reasoning, and visualization within a unified pipeline can address several longstanding challenges in LLM interpretability. Unlike prior approaches that typically examine attribution, causality, or visualization independently [5,6,8–11], the proposed framework combines these complementary perspectives within an efficiency-aware architecture, thereby addressing limitations identified in previous research.

### 6.1 Interpretation of findings

The fidelity improvements reported in Table 2 provide empirical support for prior observations that attention weights do not necessarily constitute faithful explanations of model behavior [8]. Furthermore, the results indicate that combining attribution-based and intervention-based explanations can improve alignment between generated explanations and underlying model decision processes. These findings are consistent with recent studies advocating multi-method explainability strategies for complex AI systems [13,14].

The human-centered evaluation presented in Table 3 demonstrates that the generated explanations were not only technically faithful but also more understandable and trustworthy from the perspective of end users. This observation aligns with the interpretability principles proposed by Doshi-Velez and Kim [35], who emphasize the importance of balancing technical fidelity with human comprehension. Importantly, the integration of causal reasoning improved explanation accessibility for non-expert users, thereby helping bridge the gap between AI researchers and practical stakeholders.

The efficiency analysis summarized in Table 4 shows that the proposed framework introduces only modest runtime and memory overhead (less than 25%) while delivering substantial improvements in explanation quality. Previous surveys have noted that the real-world adoption of explainable AI depends heavily on computational feasibility and practical applicability [13]. By explicitly incorporating efficiency considerations into the framework design, the proposed approach advances beyond explanation methods that focus exclusively on interpretability without accounting for resource constraints, thereby supporting the practical application of explainable AI in computationally demanding environments.

The overall fidelity score of 0.76 obtained by the proposed hybrid framework indicates that the generated explanations remain highly consistent with the underlying model predictions during insertion and deletion evaluations. Specifically, the score reflects the ability of the explanations to preserve model decision behavior when influential tokens are systematically removed or reintroduced. Compared with the baseline explanation methods evaluated in this study, the higher fidelity score demonstrates that the proposed hybrid framework more accurately captures the reasoning patterns underlying LLM predictions, thereby providing explanations that are both more faithful and more reliable for practical interpretation.

### 6.2 Practical implications

The hybrid framework offers clear pathways for practical application in high-stakes decision-support systems:

- Healthcare: Clinicians can use causal-enhanced explanations to validate diagnostic outputs and trace them back to medically relevant cues.
- Finance: Transparent reasoning chains enable auditors to evaluate models for compliance in credit risk, fraud detection, and forecasting.
- Legal systems: Unlike heuristic methods, causal-grounded explanations align with evidentiary standards [24], providing defensible rationales for automated recommendations.

By tailoring outputs to both technical and non-technical audiences, the framework bridges the research-to-practice gap, a challenge that has hindered adoption of prior XAI tools.

### 6.3 Limitations

While the proposed hybrid explainability framework demonstrates promising performance, several limitations warrant further investigation:

1. Scalability: Although the framework introduces only modest computational overhead for the evaluated models, intervention-based explainability remains computationally demanding for very large LLMs and long input sequences. As model size, sequence length, and the number of intervention operations increase, runtime overhead is expected to grow approximately linearly with sequence length and intervention count. To maintain practical computational efficiency in real-world applications, future implementations may incorporate optimization strategies such as selective layer analysis, adaptive intervention sampling, token importance pre-selection, and parallel execution of independent explanation tasks while preserving explanation fidelity.
2. Subjectivity in Human Studies: The human-centered evaluation involved a moderate sample size (n = 15), and participant ratings may be influenced by individual expertise and prior familiarity with AI systems. Although suitable for exploratory comparative evaluation, larger and more diverse participant cohorts are required to improve the generalizability and statistical robustness of human interpretability assessments.
3. Benchmark Scope: Standard benchmark datasets such as GLUE, SQuAD, and IMDB provide representative NLP evaluation tasks but may not fully capture the complexity, regulatory requirements, and domain-specific reasoning encountered in high-stakes applications such as healthcare, finance, and legal decision-making. Future work should evaluate the proposed framework using specialized domain-specific benchmarks and representative real-world application scenarios to further assess its robustness, generalizability, and practical applicability.
4. Model Versions: The experimental evaluation was conducted using GPT-3.5/4 and LLaMA-13B, which remain widely adopted, well-documented, and reproducible baselines for explainability research. Although newer models, such as GPT-4.1 and LLaMA-3, may exhibit different reasoning behaviors and internal representations, they were not included in the present study. Future research should evaluate the proposed hybrid explainability framework on newer generations of large language models, including GPT-4.1, LLaMA-3, and subsequent Transformer-based architectures, to further assess its generalizability and robustness across evolving model families.

### 6.4 Ethical and regulatory considerations

Explainability is central to trustworthy AI [22], but explanations themselves may introduce risks. For example, attribution maps could unintentionally leak sensitive training data or reveal system vulnerabilities exploitable by adversaries. Regulatory frameworks such as the EU AI Act [23] are making explainability a legal requirement in high-risk AI. Our framework’s fidelity and clarity provide a foundation for compliance, but further work is required to align outputs with dynamic standards of accountability and governance.

Our framework addresses these requirements by improving clarity and fidelity, but further work is needed to align explanations with evolving standards of legal accountability and governance. In this regard, the open challenges articulated in the XAI 2.0 manifesto [14] reinforce the importance of developing approaches that are not only technically rigorous but also interdisciplinary, bridging machine learning, human factors, and regulatory compliance.

### 6.5 Future directions

Building on these contributions, three promising directions emerge:

1. Multimodal Extension: Extending hybrid explanations to LLMs that integrate text, vision, and speech.
2. Adaptive Explanations: Customizing explanation detail dynamically to match user expertise, from clinicians to policymakers.
3. Standardized Benchmarks: Establishing shared datasets and metrics for evaluating explanation fidelity, usability, and societal impact at scale.

## 7 Conclusion

This study proposed a hybrid explainability framework for Large Language Models (LLMs) that integrates saliency-based attribution, causal reasoning, and user-oriented visualization into an efficiency-aware pipeline. Evaluations across benchmark datasets (GLUE, SQuAD, IMDB, and domain-specific corpora) and representative architectures (BERT, T5, GPT, and LLaMA) demonstrate three key outcomes: (i) higher fidelity in capturing model reasoning, (ii) improved clarity and trustworthiness in human-centered evaluations, and (iii) scalability with less than 25% computational overhead.

Unlike prior single-method approaches such as LIME, SHAP, or attention visualization, the proposed framework unifies complementary strategies into a reproducible design that balances technical rigor with practical usability. These characteristics indicate the potential suitability of the framework for practical applications in high-stakes domains, including healthcare, finance, and law, where transparency and accountability are essential.

Beyond applied contributions, the study advances theoretical discussions on explainability by reframing it as a socio-technical challenge encompassing fidelity, interpretability, efficiency, and governance. Importantly, while current experiments focus on models such as GPT-3.5/4 and LLaMA-13B, the framework is model-agnostic and can be extended to next-generation systems (e.g., GPT-4.1, LLaMA-3) and multimodal architectures. Future work will also explore adaptive explanation strategies and standardized evaluation protocols, further strengthening the responsible adoption of LLMs.
